# Supplementary material for: The impact of Public Health Emergency (PHE) on the news dissemination strength: Evidence from Chinese-Speaking Vloggers on YouTube
Source: PLoS One. 2023 Nov 29;18(11):e0294665. doi: 10.1371/journal.pone.0294665 (PMC10686435; doi:10.1371/journal.pone.0294665)
Supplement: S1 Table — (DOCX) [file pone.0294665.s001.docx]

**Table S1. Examples of Representative keywords.**

| Keywords | News content^a^ |
| --- | --- |
| Chang'e 4 | The Chang'e 4 probe landed on the far side of the moon, |
| Fuxing | The Fuxing CR200J model train officially begins service in China |
| Jiangsu chemical plant explosion | An explosion occurred at a chemical plant in Yancheng, Jiangsu, leading to at least 78 deaths |
| Belt and Road | The Second Belt and Road Forum for International Cooperation was held from 25 to 27 April 2019 in Beijing. |
| Shanghai waste management | The "Regulations on the Management of Domestic Waste in Shanghai" came into effect, announcing that Shanghai had entered the era of mandatory household waste classification. |
| 70th anniversary; military parade | China celebrates 70th anniversary with its biggest ever military parade |
| Wuhan; Huanan Seafood Market; lockdown; COVID-19; Coronavirus; Zhong Nanshan; Huoshenshan Hospital; vaccines | The COVID-19 pandemic |
| Peak carbon emissions; Carbon neutral | At the 2020 United Nations General Assembly, President Xi Jinping declared China's objective to reach peak carbon emissions before 2030 and achieve carbon neutrality by 2060. |
| China International Import Expo | The 3rd China International Import Expo was held in Shanghai. |

^a^News content is derived from the Wikipedia articles "2019 in China" and "2020 in China", documenting events in China for the years 2019 and 2020.
