# Supplementary material for: The impact of Public Health Emergency (PHE) on the news dissemination strength: Evidence from Chinese-Speaking Vloggers on YouTube
Source: PLoS One. 2023 Nov 29;18(11):e0294665. doi: 10.1371/journal.pone.0294665 (PMC10686435; doi:10.1371/journal.pone.0294665)
Supplement: Attachment — Submitted filename: Response to Reviewers.docx [file pone.0294665.s004.docx]

Dear Reviewers,

We greatly appreciate your attentive review, valuable insights, and constructive recommendations, all of which has significantly improved the presentation of our manuscript. Taking your review comments into serious consideration, we have carefully revised our manuscript. The revised portions are marked in red in the paper. The main revisions in the paper and our responses to your comments are as follows:

**Reviewer #1:**

**• Point 1: Title**: The title of the paper is not descriptive enough. Since the paper is about a specific econometric relationship, it should state so unambiguously. Which variable affects which variable?

**Response 1: The revised version:** The impact of Public Health Emergency (PHE) on the news dissemination strength: Evidence from Chinese-Speaking Vloggers on YouTube

**• Point 2:** **Abstract**: The abstract is well done but it appears to be too long. The sentence “The proposed findings … of authentic information” can be summarized by removing the numbered list (1)-(3). The abstract should summarize the introduction.

**Response 2: The revised version:** The proposed findings provide essential insights into effective news dissemination in potential future public health events.

**• Point 3: Introduction**: The introduction summarizes the whole paper by (a) stating the problem to be addressed and justifying the value of doing so, (b) specifying how the problem is or was addressed (materials and methods), (c) summarizing the results and their implications, and (d) drawing a conclusion and what it recommends for policy and further research. Some elements of these are there but are not all expressed succinctly, and they should be.

**Response 3:** Several modifications were made to the introduction: (a) some redundant content was removed for a more concise introduction; (b) more literature that is closely related to our research was introduced, specifying how the problem was addressed; (c) a brief overview of the investigated problem was provided; (d) the detailed literature review was moved to the "Research background and relevant literature" section.

**The revised version:**

Recently, there have been frequent public health emergencies, causing substantial adverse influences on public health. In particular, the outbreak of COVID-19 in 2019 triggered a significant number of infections and deaths on a global level. By the end of July 2023, the World Health Organization (WHO) reports that there have been over 768 million confirmed cases of COVID-19 across the world, with 6.95 million reported deaths [1]. In addition to the direct effect of the virus on public health, the preventive measures incorporated to control the pandemic, such as lockdown and social distancing, have disrupted the normal social gatherings and interactions, leading to higher levels of sleep disorders, anxiety, stress, and depression, consequently contributing to a rise in mental health concerns [2-4]. Moreover, the COVID-19 pandemic has intensively strained the healthcare systems around the globe, thereby stimulating not only resource shortages but also negatively affecting the medical services for non-COVID-19 patients [5, 6]. At the same time, certain patients may have postponed seeking medical care during the pandemic due to concerns about virus infection, thus resulting in the neglect and deterioration of other health matters [7]. Owing to the severe negative impacts of public health emergencies on public health, the public actively seeks information about these events through various channels with the expectation of quickly understanding the situation to protect their own health, as well as that of their family and friends [8-9]. Therefore, the effective dissemination of information to the public through media during the early stages of a public health emergency emerges as a topic of shared concern for both policymakers and research scholars.

News media play a vital role in disseminating public health and policy information during public health emergencies [10-14]. This allows the relevant authorities to instantly share the latest updates, guidelines, and safety precautions with the general public, consequently aiding in controlling diseases and informing the public regarding the latest developments [15]. In fact, news media not only convey potential risks but also influence public perceptions through the presented content, volume, and tone of media coverage [16, 17]. Furthermore, through social media channels, the news sources not only effectively disseminate information but also promptly receive feedback from audiences, extending a framework for prevalent public debates concerning policy responses, including conflicting priorities related to the timing or stringency of policy implementation [11, 12]. Besides this, news reports swiftly disseminate treatment protocols during public health emergencies at regional-, national-, and international levels [18]. This supports less-resourced facilities to quickly adopt protocols and incorporate essential adjustments to fit their distinct circumstances and accessible resources. Furthermore, news coverage facilitates isolated individuals to comprehend external information. As a result, news coverage mitigates the sense of isolation and boredom connected with anxiety, depression, and long-term distress, thus making it an essential resource for isolation at home to reduce the psychological impact [19]. Evidently, the significant influence of news dissemination is undeniable during public health emergencies, as witnessed during the COVID-19 pandemic, where news media played an imperative role in broadcasting information, influencing billions worldwide [20].

With the development of Information and Communication Technology, significant changes have taken place in the way and nature of news dissemination. Many scholars have systematically studied the characteristics of news dissemination during public health emergencies. First, time factors are considered as important factors affecting the news dissemination during public health emergencies [21]. In different life cycles, there are great differences in the propagation speed and diffusion volumes of event-related information on social media [22, 23]. Second, media factors are also significant factors influencing the news dissemination during public health emergencies. The scope and diffusion degree of news on social media vary significantly based on the media type and its influence [24-25]. Additionally, the contents of news are also important influence factors for news dissemination in public health emergencies. For instance, when public health emergencies occur, there are often great differences in the dissemination of news under different topic categories on social media [26, 27]. Similarly, for information with different sentiments and emotions, the dissemination of information presents different trends and states [28].

Though there is a consistent interest from different disciplines, studies on the general characteristics of news dissemination during public health emergencies demonstrate certain limitations. First, most existing studies have explored the general characteristics of news dissemination in public health emergencies through descriptive statistics, while causal analysis is relatively lacking [21]. Second, most research studies on news dissemination have primarily concentrated on English-speaking audiences or China's internet users, with relatively limited literature on overseas Chinese-speaking audiences and communities. Third, research data adopted in pertinent empirical studies frequently confront challenges associated with accuracy and efficacy. For instance, non-random sampling methods and small sample sizes can lead to survivor bias, selection bias, or other methodological drawbacks.

In order to bridge the research gap discussed above, this study aims to explore the impact of Public Health Emergency (PHE) on the news dissemination strength. Specifically, to achieve this goal, this research study adopted big data from Chinese-speaking channels on YouTube while employing the difference-in-differences (DiD) method to analyze the dynamic changes in the news dissemination strength of pertinent content during public health emergencies. In addition, this paper explored the heterogeneity in changes in news dissemination strength across different types of video bloggers. YouTube serves as the largest video-sharing website on the internet by hosting a plethora of video bloggers who share different types of videos. This platform also attracts a massive audience that watches video content and extends personal comments. As of Jan 2022, YouTube had reached 2.562 billion monthly active users across the world [29]. In Taiwan, Hong Kong, and countries/regions where overseas Chinese populations exceed 1 million, the proportion of YouTube users among the population aged 18 and above is remarkably high [30]. Therefore, the researchers believe that Chinese-speaking channels on YouTube serve as a widely engaged online social platform for overseas Chinese, Hong Kong, and Taiwan residents, with its micro-level data carrying strong representativeness. Thus, research undertaken on this platform can offer valuable insights into the common/general patterns of online news dissemination among overseas Chinese-speaking populations and communities.

This research article contributes to the extant literature in the following manners. Firstly, a comprehensive analysis of the changes in news dissemination strength of online media during public health emergencies is performed in this study using the difference-in-differences (DiD) approach and event study. In fact, the DiD approach and event study are commonly applied data analysis methods in the field of econometrics, which make the study results not only hold statistical significance but also extend explicit insights into the causal relationships between public health emergency and the variations in dissemination strength of relevant news. Secondly, this research analyzes the news dissemination of Chinese-speaking channels on YouTube during public health emergencies. The potential audience of these Chinese-speaking channels primarily includes Chinese-speaking internet users outside of mainland China. By examining the dissemination of relevant news among overseas Chinese-speaking communities during public health emergencies, this research paper offers a valuable supplement to the research on the general characteristics of news dissemination. Thirdly, this paper employs big data generated through random sampling in order to study the dynamic effects on the dissemination strength of relevant news content during public health emergencies. As a consequence, the abundant data resources enable the researchers to carry out multifaceted analyses. In specific, this study not only scrutinizes the impact of public health emergencies on the dissemination strength of relevant news but also ascertains the potential heterogeneity of the proposed impact among various types of YouTube channels, such as lifestyle bloggers, and news & political bloggers, institutional and personal media, and local and foreign media.

**• Point 4: Research Background and Methodology**: This section needs serious consideration. In fact, it may be best to change it to Research Background and Relevant Literature, i.e., 2.1 Research Background and 2.2 Relevant Literature. The background is clearly necessary to frame the PHE in China, but just as clearly the reader would want to see the positioning of the paper in the existing literature. What is missing from the extant literature and how does this paper fill the gap? I’d then move the Methodology to the next section.

**Response 4:** We change this section to “Research background and relevant literature,” which includes “Research background” and “Relevant literature.” Relevant literature was reviewed to illustrate the positioning of the paper within the existing literature.

**The revised version:**

**Relevant literature**

During the outbreak of COVID-19, social media became an important channel for the government to disseminate information to the public [33]. Most relevant studies primarily explored the characteristics of news dissemination during public health emergencies from the perspectives of time factors and media factors.

**Time factors affecting news dissemination during public health emergencies**

Public health emergencies do not emerge abruptly; they progress through a series of different stages and form different life cycles [34]. Similarly, the dissemination dynamics of event-related news on social media exhibit variations across different life cycle stages [21, 35]. Concerning the correlation between news dissemination strength and the life cycle stages of public health emergencies, different studies have yielded diverse conclusions. Research specifically related to the COVID-19 event found that the dissemination strength of news on social media aligns with the trend of the COVID-19 outbreak in the real world [33]. However, studies with a differing view argue that with the rapid development and extensive application of the internet, news related to emergency events spread faster than ever before [36]. As a result, the news dissemination of public health emergencies generally demonstrate a shorter life cycle, and a briefer active period compared to the trend of real-life crisis events [37].

Moreover, there also exists a lack of sustained news coverage during public health emergencies. Regardless of the prevalent threats to public health, news coverage promptly dwindled succeeding the initial declaration of a public health emergency [16, 38]. In fact, similar challenges in terms of media coverage were observed during the 2014 Ebola epidemic and the 2003 Severe Acute Respiratory Syndrome (SARS) outbreak [39]. This implies that despite the persistence of related risks and challenges, the public may misconstrue the urgency and significance of the matter, owing to the lack of persistent media coverage. The stated drawbacks remind the researchers that there is a need to further comprehend and explore the role of media during public health emergencies as these measures shall prove to be essential tools for combating future possible outbreaks.

To further understand the time factors affecting news dissemination during public health emergencies, this research examines the dynamic trends of news dissemination strength during the COVID-19 outbreak. This not only supplements to the previous research of the general characteristics of news dissemination during public health emergencies, but also further explores the reasons for the lack of persistence in news reporting during such events.

**Media factors affecting news dissemination during public health emergencies**

During public health emergencies, information published by different media types varies greatly in the scope of influence and degree of diffusion on social media [24]. On the one hand, the influence of media themselves is an important factor affecting news dissemination. The number of subscribers a media has serves as the most intuitive standard for measuring their influence, and media outlets with a large number of subscribers tend to have a wider reach and diffusion for their published news [24, 25, 40). For instance, during public health emergencies, news dissemination strength varies among news media and celebrities, media organizations and ordinary individuals, celebrities and ordinary individuals [41, 42]. On the other hand, the media's credibility and communication abilities significantly influence news dissemination during health emergencies. For example, a Twitter study suggested that during COVID-19, news from "Mainstream or Local News" sources had greater dissemination strength than "Government or Public Health" sources [43]. Therefore, government media can enhance their news dissemination effectiveness for public health emergencies by strengthening their credibility. Similarly, public health organizations need to improve their information dissemination capacity through better alignment with the general public’s information needs during public health crises [44, 45].

Although the influence of media types on news dissemination has attracted the attention of scholars, most of the previous studies were limited by insufficient data sources and were unable to make a more comprehensive analysis of media with different attribute characteristics. To address the aforementioned research gap, in the heterogeneity analysis section of this research article, media types are categorized as “news and political bloggers” and “lifestyle bloggers” from the perspective of content. Similarly, based on attribute characteristics of media, media types are classified as “local media” and “foreign media,” “individual media” and “institutional media.” This study subsequently examines the differences in news dissemination by different types of media during public health emergencies. This work not only complements previous research but also further unveils the general characteristics of news dissemination during public health emergencies.

**• Point 5: Data Source and Processing**: The data and methodology belong together (materials and methods). So, this section should be simply Data and Methodology: 3.1 Data Source, 3.2 Data Processing, 3.3 Summary Stats (I don’t think a subsection is needed for this), and 3.4 Methodology. Under the methodology all variables and parameters should be defined, conventionally from left to right. From Equation 1 I noted that X is not defined and the expectations about the interaction term “post COVID19News” are not characterized. Later in the paper it sounds like X are control variables, but no examples are given -- at least a few should be provided. Also, equations are sentences and correct punctuation applies to them, just as it applies to ordinary sentences.

**Response 5:** This section was revised to "Data Processing and Research Methodology," comprising subsections 3.1 Data collection, 3.2 Data Processing, and 3.3 Research Methodology. All variables and parameters were defined, and the expectations regarding the coefficient of the interaction term (β_3_) were characterized. Furthermore, some examples of control variables (X’) are introduced. Finally, equation (1) was modified.

**The revised version:**

$Y_{ijtl}=\beta_{0}+\beta_{1}\cdot{post}_{i}+\beta_{2}\cdot{COVID-19 News}_{ijtl}+\beta_{3}\cdot\left( {COVID-19 News}_{ijtl}\cdot{post}_{i} \right)+\beta_{4}\cdot X_{ijtl}^{'}+u_{j}+u_{t}+u_{l}+\varepsilon_{ijdl}$	(1)

… Moreover, X’_ijtl_ represents control variables, which contain information such as the number of subscribers for the video bloggers, video channels/blogger location, language (Simplified Chinese/Traditional Chinese), and years of media operation.

…Moreover, the coefficient of interest, symbolized as β_3_, presents the mean treatment effect generated by the event impact. In this paper, the proposed coefficient indicates the effect of COVID-19’s public health emergency on the dissemination strength of pandemic-relevant video content, which is expected to be positive as the number of people using social media to collect information increases in face of public health emergency.

**• Point 6: Results and Discussion**: These two sections are in order, although the heterogeneity analysis appears out of nowhere; it was not motivated earlier on. What brought it on – why was it important to carry out? Is it a part of the test for robustness or separate?

**Response 6:** Heterogeneity analysis is an independent section in this research paper. **In 2.2 Relevant Literature, we highlighted the significance of heterogeneity analysis:** Although the influence of media types on news dissemination has attracted the attention of scholars, most of the previous studies were limited by insufficient data sources and were unable to make a more comprehensive analysis of media with different attribute characteristics. To address the aforementioned research gap, in the heterogeneity analysis section of this research article, media types are categorized as “news and political bloggers” and “lifestyle bloggers” from the perspective of content. Similarly, based on attribute characteristics of media, media types are classified as “local media” and “foreign media,” “individual media” and “institutional media.” This study subsequently examines the differences in news dissemination by different types of media during public health emergencies. This work not only complements previous research but also further unveils the general characteristics of news dissemination during public health emergencies.

**• Point 7: Conclusion and Policy implications**: This one is the strongest section of the paper in terms of presentation. The only question it raises is why the conclusion does not have any implications for future research. Should the reader conclude this case is open and shut?

**Response 7: The revised version:** This article highlights crucial research findings that offer insights into how health organizations can effectively disseminate relevant information in upcoming public health emergencies. Nonetheless, this study possesses inherent limitations. For instance, our data utilization is confined. Specifically, the empirical analysis of this study relied exclusively on text, neglecting potential insights from videos and images. Videos and images, intuitively, may contain richer information, suggesting avenues for further research.

**• Point 8: References**: 44 pieces of literature form the basis for this research, and I want to see how they coalesce into the big theme of the paper; this is one of the reasons why I recommend a brief literature review: What is known about this topic? What are the gaps, and what does this effort contribute to closing the gap?

**Response 8:** The references section has been updated. In refining the introduction, unrelated literature was eliminated, while more pertinent references were incorporated. Additionally, within the “Introduction” and "Relevant literature" sections, we highlighted existing research gaps and delineated this article's positioning to the current literature.

**Research gaps:**

(Introduction) Though there is a consistent interest from different disciplines, studies on the general characteristics of news dissemination during public health emergencies demonstrate certain limitations. First, most existing studies have explored the general characteristics of news dissemination in public health emergencies through descriptive statistics, while causal analysis is relatively lacking [21]. Second, most research studies on news dissemination have primarily concentrated on English-speaking audiences or China's internet users, with relatively limited literature on overseas Chinese-speaking audiences and communities. Third, research data adopted in pertinent empirical studies frequently confront challenges associated with accuracy and efficacy. For instance, non-random sampling methods and small sample sizes can lead to survivor bias, selection bias, or other methodological drawbacks.

(Relevant literature) Although the influence of media types on news dissemination has attracted the attention of scholars, most of the previous studies were limited by insufficient data sources and were unable to make a more comprehensive analysis of media with different attribute characteristics.

**This article's positioning to the current literature:**

(Introduction) This research article contributes to the extant literature in the following manners. Firstly, a comprehensive analysis of the changes in news dissemination strength of online media during public health emergencies is performed in this study using the difference-in-differences (DiD) approach and event study. In fact, the DiD approach and event study are commonly applied data analysis methods in the field of econometrics, which make the study results not only hold statistical significance but also extend explicit insights into the causal relationships between public health emergency and the variations in dissemination strength of relevant news. Secondly, this research analyzes the news dissemination of Chinese-speaking channels on YouTube during public health emergencies. The potential audience of these Chinese-speaking channels primarily includes Chinese-speaking internet users outside of mainland China. By examining the dissemination of relevant news among overseas Chinese-speaking communities during public health emergencies, this research paper offers a valuable supplement to the research on the general characteristics of news dissemination. Thirdly, this paper employs big data generated through random sampling in order to study the dynamic effects on the dissemination strength of relevant news content during public health emergencies. As a consequence, the abundant data resources enable the researchers to carry out multifaceted analyses. In specific, this study not only scrutinizes the impact of public health emergencies on the dissemination strength of relevant news but also ascertains the potential heterogeneity of the proposed impact among various types of YouTube channels, such as lifestyle bloggers, and news & political bloggers, institutional and personal media, and local and foreign media.

(Relevant literature) To further understand the time factors affecting news dissemination during public health emergencies, this research examines the dynamic trends of news dissemination strength during the COVID-19 outbreak. This not only supplements to the previous research of the general characteristics of news dissemination during public health emergencies, but also further explores the reasons for the lack of persistence in news reporting during such events.

(Relevant literature) …To address the aforementioned research gap, in the heterogeneity analysis section of this research article, media types are categorized as “news and political bloggers” and “lifestyle bloggers” from the perspective of content. Similarly, based on attribute characteristics of media, media types are classified as “local media” and “foreign media,” “individual media” and “institutional media.” This study subsequently examines the differences in news dissemination by different types of media during public health emergencies. This work not only complements previous research but also further unveils the general characteristics of news dissemination during public health emergencies.

**• Point 9: General**: The English of the paper should be corrected: Conciseness, mechanics, etc. I see a lot of typos, but the incorrect use of the semi-colon (;) is most prevalent.

**Response 9:** The manuscript's phrasing and structure have been refined for greater clarity and brevity. Moreover, we addressed spelling errors and specifically amended the incorrect use of the semi-colon (;).

**Reviewer #2:**

**• Point 1:** Introduction, Paragraph 5 line 5- authors briefly describes their study findings. I suggest removing information related to findings.

**Response 1:** we have excluded the research findings from the Introduction. Concurrently, following your suggestion in Point 4, we have relocated the first paragraph from the Research Methodology section to this segment. **The revised version is as follows**:

In order to bridge the research gap discussed above, this study aims to explore the impact of Public Health Emergency (PHE) on the news dissemination strength. Specifically, to achieve this goal, this research study adopted big data from Chinese-speaking channels on YouTube while employing the difference-in-differences (DiD) method to analyze the dynamic changes in the news dissemination strength of pertinent content during public health emergencies. In addition, this paper explored the heterogeneity in changes in news dissemination strength across different types of video bloggers. YouTube serves as the largest video-sharing website on the internet by hosting a plethora of video bloggers who share different types of videos. This platform also attracts a massive audience that watches video content and extends personal comments. As of Jan 2022, YouTube had reached 2.562 billion monthly active users across the world [29]. In Taiwan, Hong Kong, and countries/regions where overseas Chinese populations exceed 1 million, the proportion of YouTube users among the population aged 18 and above is remarkably high [30]. Therefore, the researchers believe that Chinese-speaking channels on YouTube serve as a widely engaged online social platform for overseas Chinese, Hong Kong, and Taiwan residents, with its micro-level data carrying strong representativeness. Thus, research undertaken on this platform can offer valuable insights into the common/general patterns of online news dissemination among overseas Chinese-speaking populations and communities.

**• Point 2:** Introduction, Last paragraph line 3….typographical error *this should be sample size

**Response 2: The revised version:** As a consequence, the abundant data resources enable the researchers to carry out multifaceted analyses.

**• Point 3:** **Research Background and Methodology,** 2.1 The COVID-19 Outbreak-Background information on the COVID-19 Outbreak should be summarized to be as brief as possible and moved into the introduction section so this section can be reserved for the methodology of the research.
**Response 3:** We change this section to “2. Research background and relevant literature,” which includes “2.1 Research background” and “2.2 Relevant literature.” The background information of the COVID-19 Outbreak primarily serves to frame the PHE in China. Meanwhile, Relevant literature was reviewed to illustrate the positioning of the paper within the existing literature. Moreover, we have shifted the "Research methodology" to the subsequent section, which, in conjunction with "3.1 Data collection" and "3.2 Data processing," now forms the "3. **Data processing and research methodology**" section.

**• Point 4:** 2. Research Methodology, Paragraph 1 should be moved to the introduction section.
**Response 4:** Paragraph 1 of Research Methodology has been moved to the introduction section. The revised version is as follows: YouTube serves as the largest video-sharing website on the internet by hosting a plethora of video bloggers who share different types of videos. This platform also attracts a massive audience that watches video content and extends personal comments. As of Jan 2022, YouTube had reached 2.562 billion monthly active users across the world [29]. In Taiwan, Hong Kong, and countries/regions where overseas Chinese populations exceed 1 million, the proportion of YouTube users among the population aged 18 and above is remarkably high [30]. Therefore, the researchers believe that Chinese-speaking channels on YouTube serve as a widely engaged online social platform for overseas Chinese, Hong Kong, and Taiwan residents, with its micro-level data carrying strong representativeness. Thus, research undertaken on this platform can offer valuable insights into the common/general patterns of online news dissemination among overseas Chinese-speaking populations and communities.

**• Point 5:** 3 data source and processing, Paragraph 3 line 2. The authors state that the approach for building the sampling frame of YouTube video bloggers was established by repetitively searching for top keywords from top news in mainland China. Kindly include what these top keywords are in the manuscript.

**Response 5:** We have listed examples of top keyword in Table S1 and provided indications within the main text. **The revised version:** Correspondingly, examples of these keywords can be found in S1 Table.

Table S1. Examples of Representative keywords.

| Keywords | News content^a^ |
| --- | --- |
| Chang'e 4 | The Chang'e 4 probe landed on the far side of the moon, |
| Fuxing | The Fuxing CR200J model train officially begins service in China |
| Jiangsu chemical plant explosion | An explosion occurred at a chemical plant in Yancheng, Jiangsu, leading to at least 78 deaths |
| Belt and Road | The Second Belt and Road Forum for International Cooperation was held from 25 to 27 April 2019 in Beijing. |
| Shanghai waste management | The "Regulations on the Management of Domestic Waste in Shanghai" came into effect, announcing that Shanghai had entered the era of mandatory household waste classification. |
| 70th anniversary; military parade | China celebrates 70th anniversary with its biggest ever military parade |
| Wuhan; Huanan Seafood Market; lockdown; COVID-19; Coronavirus; Zhong Nanshan; Huoshenshan Hospital; vaccines | The COVID-19 pandemic |
| Peak carbon emissions; Carbon neutral | At the 2020 United Nations General Assembly, President Xi Jinping declared China's objective to reach peak carbon emissions before 2030 and achieve carbon neutrality by 2060. |
| China International Import Expo | The 3rd China International Import Expo was held in Shanghai. |

^a^News content is derived from the Wikipedia articles "2019 in China" and "2020 in China", documenting events in China for the years 2019 and 2020.

**• Point 6:** Paragraph 3 line 12. The authors state that small samples were excluded due to limited influence and a minimum subscriber threshold was set. Kindly state the what this threshold limit is and a justification for choosing it if possible.

**Response 6:** We explained the reason for setting a minimum subscriber threshold in Paragraph (5). Additionally, in the robustness check section, we tested different minimum subscriber thresholds, and the results were consistent with the baseline regression.

(Data collection) (5) The samples with fewer than 10,000 subscribers are excluded from the sampling base. Primarily, this exclusion serves to resolve the concern of non-random sampling resulting from selective content distribution on YouTube. From the viewpoint of YouTube's content distribution mechanism, there exists a strong selective content distribution when promoting videos from smaller channels. Moreover, in the section on robustness analysis, regression results are also provided based on the samples with subscribers greater than or equal to 50,000 and 1,000 subscribers, respectively.

(Robustness test) Firstly, various samples are created based on different thresholds of subscription count. Previously, among all Chinese-speaking video bloggers closely linked to mainland China, a sample with a subscription count of 10,000 or more was chosen by the authors. At present, the lower limit for sample subscription counts has been adjusted to 1,000 or 50,000, and the corresponding regression results are presented in Table 3 (a) and (b), respectively. Furthermore, the sampling timeframe remains consistent for both scenarios, spanning from 31st Dec 2019 to 20th Feb 2020. It is evident that all interaction term coefficients are statistically significant, whereas the magnitudes and directions of the coefficients are the same as those in Table 2.

**• Point 7:** Paragraph 4 states that video bloggers whose published videos contain 10% or more content relevant topics of mainland China were selected. Kindly explain what constitutes “content relevant topics of mainland China”.

**Response 7: This statement has been modified to:** Specifically, through text analysis of video titles, descriptions, and tags, video bloggers who had 10% or more of their videos related to mainland China topics were selected. Additionally, in the "Data processing" section, we further introduced the methods used to determine whether the video content is related to mainland China topics as follows:

In this study, video content is identified based on video titles, descriptions, and tags. For this purpose, the "Jieba" Chinese text segmentation module is employed to perform word segmentation on the required text content of the videos. Subsequently, this segmented text is matched with the predefined "Mainland China Corpus" and "COVID-19 PHE Corpus", in order to identify videos related to mainland China and the COVID-19 Public Health Emergency (PHE). For instance, the title is decomposed by the accurate mode of the "jieba" word segmentation into words such as "China," "succeeded in," "CDC," "isolating," "the," "first," "novel coronavirus strain," etc., when a video title is "China CDC succeeded in isolating the first novel coronavirus strain". Afterward, this title is matched with the vocabulary from the two aforementioned corpora. As a result, words such as "China" and "novel coronavirus strain" are successfully matched with existing terms in the "Mainland China Corpus" and "COVID-19 Epidemic Corpus" respectively. Consequently, such a video is characterized as both relevant to mainland China topics and linked to the COVID-19 pandemic subject.

**• Point 8:** Summary statistics, Paragraph 1- The authors state that “the number of likes on a video represents the audience's level of liking or agreement with the published content, as well as their confidence in the content, source, and channel” This is subjective however, if this measure has been used in similar research the authors should clearly indicate this and provide adequate references.

**Response 8:** To avoid overly subjective interpretation, we have revised the statement to: "The number of likes on a video represents the audience's level of appreciation for the published content."

We earnestly appreciate your work and hope that our revisions live up to your expectation. Again, thank you very much for your comments and suggestions.
