# Supplementary material for: The impact of Public Health Emergency (PHE) on the news dissemination strength: Evidence from Chinese-Speaking Vloggers on YouTube
Source: PLoS One. 2023 Nov 29;18(11):e0294665. doi: 10.1371/journal.pone.0294665 (PMC10686435; doi:10.1371/journal.pone.0294665)
Supplement: Attachment — Submitted filename: Referee report for PLOS ONE News Dissemination.docx [file pone.0294665.s003.docx]

**Referee’s Report**

**Title**: News Dissemination in Video-Sharing Platform During Public Health Emergency (PHE): Evidence from Chinese-Speaking Vloggers on YouTube

**Authors**: Dan Sun and Guochang Zhao

**Manuscript Number**: PONE-D-23-29289

**Journal**: PLOS ONE

**Overview:** This paper assesses the impact of the national public health emergency (PHE) on the spread of news. It uses Covid19 in China as the emergency, and models it in a difference-in-difference (DiD) econometric environment with data processed from video-sharing platforms on YouTube in terms of views, likes, and comments. The study finds that during the PHE views, likes, and comments increased rapidly in the first week and slowly declined by the fourth week. Different vloggers had different impacts, but the effect of videos was larger than that of traditional news sources, and subscribers tended to continue their subscriptions even after the PHE had passed. The concluding policy implications of the paper are clear, and they recommend that the government use vloggers to disseminate PHE, and in some instances even collaborate with them.

The paper deals with a topic of global importance; climate change will likely increase emergencies and hence PHEs. Under such conditions fast and effective news dissemination will be needed to facilitate adaptation and resilience (economic, risk, and supply chain). While the paper is a great project, below I describe areas that would benefit from additional consideration.

**Title**: The title of the paper is not descriptive enough. Since the paper is about a specific econometric relationship, it should state so unambiguously. Which variable affects which variable?

**Abstract**: The abstract is well done but it appears to be too long. The sentence “The proposed findings … of authentic information” can be summarized by removing the numbered list (1)-(3). The abstract should summarize the introduction.

**Introduction**: The introduction summarizes the whole paper by (a) stating the problem to be addressed and justifying the value of doing so, (b) specifying how the problem is or was addressed (materials and methods), (c) summarizing the results and their implications, and (d) drawing a conclusion and what it recommends for policy and further research. Some elements of these are there but are not all expressed succinctly, and they should be.

**Research Background and Methodology**: This section needs serious consideration. In fact, it may be best to change it to Research Background and Relevant Literature, i.e., 2.1 Research Background and 2.2 Relevant Literature. The background is clearly necessary to frame the PHE in China, but just as clearly the reader would want to see the positioning of the paper in the existing literature. What is missing from the extant literature and how does this paper fill the gap? I’d then move the Methodology to the next section.

**Data Source and Processing**: The data and methodology belong together (materials and methods). So, this section should be simply Data and Methodology: 3.1 Data Source, 3.2 Data Processing, 3.3 Summary Stats (I don’t think a subsection is needed for this), and 3.4 Methodology. Under the methodology all variables and parameters should be defined, conventionally from left to right. From Equation 1 I noted that X is not defined and the expectations about the interaction term “post COVID19News” are not characterized. Later in the paper it sounds like X are control variables, but no examples are given -- at least a few should be provided. Also, equations are sentences and correct punctuation applies to them, just as it applies to ordinary sentences.

**Results and Discussion**: These two sections are in order, although the heterogeneity analysis appears out of nowhere; it was not motivated earlier on. What brought it on – why was it important to carry out? Is it a part of the test for robustness or separate?

**Conclusion and Policy implications**: This one is the strongest section of the paper in terms of presentation. The only question it raises is why the conclusion does not have any implications for future research. Should the reader conclude this case is open and shut?

**References**: 44 pieces of literature form the basis for this research, and I want to see how they coalesce into the big theme of the paper; this is one of the reasons why I recommend a brief literature review: What is known about this topic? What are the gaps, and what does this effort contribute to closing the gap?

**General**: The English of the paper should be corrected: Conciseness, mechanics, etc. I see a lot of typos, but the incorrect use of the semi-colon (;) is most prevalent.

Having said all that, this is a great project – good luck with it!
